# Inhibition of the glutamate-cysteine ligase catalytic subunit with buthionine sulfoximine enhances the cytotoxic effect of doxorubicin and cyclophosphamide in Burkitt lymphoma cells

**DOI:** 10.1007/s13353-023-00797-1

**Published:** 2023-11-02

**Authors:** Marta Kazimierska, Aleksandra Leśniewska, Anja Bakker, Arjan Diepstra, Marta Elżbieta Kasprzyk, Marta Podralska, Karolina Rassek, Joost Kluiver, Anke van den Berg, Natalia Rozwadowska, Agnieszka Dzikiewicz-Krawczyk

**Affiliations:** 1 Institute of Human Genetics, Polish Academy of Sciences, Poznań, Poland; 2 Institute of Bioorganic Chemistry, Polish Academy of Sciences, Poznań, Poland; 3 Department of Pathology & Medical Biology, University of Groningen, University Medical Centre Groningen, Groningen, The Netherlands

**Keywords:** Burkitt lymphoma, Glutathione, GCLC, Buthionine sulfoximine, Cancer therapy

## Abstract

Burkitt lymphoma (BL) is a highly aggressive lymphoma that mainly affects children and young adults. Chemotherapy is effective in young BL patients but the outcome in adults is less satisfactory. Therefore, there is a need to enhance the cytotoxic effect of drugs used in BL treatment. Glutathione (GSH) is an important antioxidant involved in processes such as regulation of oxidative stress and drug detoxification. Elevated GSH levels have been observed in many cancers and were associated with chemoresistance. We previously identified GCLC, encoding an enzyme involved in GSH biosynthesis, as an essential gene in BL. We now confirm that knockout of GCLC decreases viability of BL cells and that the GCLC protein is overexpressed in BL tissues. Moreover, we demonstrate that buthionine sulfoximine (BSO), a known inhibitor of GCLC, decreases growth of BL cells but does not affect control B cells. Furthermore, we show for the first time that BSO enhances the cytotoxicity of compounds commonly used in BL treatment, doxorubicin, and cyclophosphamide. Given the fact that BSO itself was not toxic to control cells and well-tolerated in clinical trials, combination of chemotherapy with BSO may allow reduction of the doses of cytotoxic drugs required to obtain effective responses in BL patients.

**Supplementary Information:**

The online version contains supplementary material available at 10.1007/s13353-023-00797-1.

## Introduction

Burkitt lymphoma (BL) is a highly aggressive B-cell non-Hodgkin lymphoma. A translocation involving the c-*MYC* gene locus on chromosome 8 is the hallmark of BL and occurs in approximately 95% cases (Kalisz et al. 2019). Three clinical variants can be distinguished in BL: endemic (associated with EBV infection), sporadic, and immunodeficiency-related (Bellan et al. 2003). Treatment outcome depends on the patient’s age and disease stage. Cure rate for sporadic BL approaches 90% in children and young adults from developed countries. However, standard chemotherapy regimens are often insufficient for treatment of adult BL patients. In addition, chemotherapy leads to severe side effects in a substantial proportion of the patients (Bellan et al. 2003). Therefore, less toxic treatment options are needed.

A characteristic feature of cancer cells is the reprogramming of metabolism to meet their increased energy demand. Cancer cells are often characterized by high glucose uptake, lactate production, and glycolytic metabolism. In addition, increased synthesis of nucleotides, amino acids, and fatty acids supports growth and proliferation of malignant cells. However, as a by-product of enhanced metabolism, cancer cells face high levels of reactive oxygen species and need to mitigate oxidative stress (Dang 2012).

Glutathione (GSH) is a tripeptide, γ-L-glutamyl-L-cysteinylglycine, present in all mammalian tissues. It is a potent antioxidant involved in the maintenance of redox balance in cells (Traverso et al. 2013). GSH is essential for detoxification of xenobiotics or products of oxidative stress (Desideri et al. 2019) and is involved in DNA repair, cell proliferation, and ferroptosis (Kennedy et al. 2020). GCLC (glutamate-cysteine ligase catalytic subunit) together with GCLM (glutamate-cysteine ligase modifier subunit) forms the γ-glutamylcysteine synthetase (GCL), a rate-limiting enzyme involved in the first step of *de novo* GSH biosynthesis (Griffith and Mulcahy 1999). Increased GCLC levels have been observed in several cancers, and high GCLC expression has been associated with drug resistance (Fujimori et al. 2004; Hiyama et al. 2018; Jarvinen et al. 2002). It was shown that inhibition of GSH enhances the effect of drug treatment, e.g., in neuroblastoma (O’Dwyer et al. 1996; Villablanca et al. 2016).

Buthionine sulfoximine (BSO) is a specific GCLC inhibitor capable of reducing GSH levels *in vitro* and *in vivo* (Bailey 1998; Griffith and Meister 1979). BSO enhanced the anti-cancer potential of drugs and active components in breast cancer, neuroblastoma, and lymphoma (Dusre et al. 1989; Marengo et al. 2008; Yang et al. 2010). Studies in mice demonstrated that BSO is well-tolerated and non-toxic (Dorr et al. 1986; Ishikawa et al. 1988). Clinical trials in neuroblastoma indicated that BSO enhanced treatment efficiency of melphalan (O’Dwyer et al. 1996; Villablanca et al. 2016). These properties highlight the potential use of BSO in anti-cancer therapy.

So far, the role of enzymes involved in GSH synthesis and the therapeutic potential of their inhibition have not been investigated in B-cell lymphoma. In our previous study, we conducted a high-throughput genome-wide CRISPR/Cas9 screen in the ST486 BL cell line (Niu et al. 2020) (Kazimierska et al. 2023). Genes encoding all three enzymes involved in GSH synthesis were found to be essential for growth of ST486 cells. *GCLC* and *GSS* (glutathione synthetase) (but not *GCLM*) were also essential in BL41, BJAB, and Jijoye cell lines in other studies (Panea et al. 2019; Wang et al. 2015). Analysis of genetic dependencies available from depmap.org revealed that genes involved in GSH synthesis are essential exclusively for blood cancers: acute lymphoblastic leukemia and B-cell lymphoma (Supplementary Figure 1). Since a specific inhibitor was available for GCLC, we focused on this protein and studied the effect of GCLC inhibition with BSO alone or in combination with commonly used chemotherapeutics on survival of BL cells.

## Materials and methods

### Cell lines

BL cell lines and B-cell lymphoblastoid cell lines (K1-K4) were cultured in Roswell Park Memorial Institute 1640 medium (Lonza, Basel, Switzerland) supplemented with 2 mM L-glutamine (Biowest, Nuaille, France), 1% penicillin/streptomycin (Biowest), and 10–20% fetal bovine serum (FBS) (Sigma-Aldrich, Saint Louis, MO, US) in a 5% CO_2_ incubator at 37 °C. HEK293T (DSMZ, Braunschweig, Germany) used for lentiviral particle production was cultured in low glucose Dulbecco’s Modified Eagle’s Medium (Lonza) supplemented as described above. DG75, BL14, and CA46 cell lines were obtained from DSMZ, ST486 cell line from ATCC (Manassas, VA, US) and K1-K4 EBV-transformed lymphoblastoid cell lines were established from healthy donors in our lab (Dzikiewicz-Krawczyk et al. 2012). Briefly, peripheral blood lymphocytes were isolated and resuspended in RPMI-1640 medium supplemented with 20% FBS, 2 μg/ml cyclosporin A, and 10% Epstein-Barr virus containing medium. Cells were maintained in the standard conditions. After occurrence of the clumps, cells were cultured in medium supplemented with 15% FBS.

### Immunohistochemistry staining

Fourteen primary formalin-fixed paraffin-embedded (FFPE) BL tissues and 3 tonsil samples were selected from the tissue repository of the Pathology and Medical Biology department of the University Medical Center Groningen. Tissue was used in accordance with the Declaration of Helsinki, and the protocol was approved by the Medical Ethical Review Board of the UMCG (RR#201800554). Immunohistochemistry was conducted according to standard protocols using 10 mM citrate buffer (pH 6.0) in the microwave for antigen retrieval. GCLC was detected using an anti-GCLC antibody from Abcam (ab53179, dilution 1:800, Cambridge, United Kingdom). Visualization was performed with diaminobenzidine, and slides were assessed by an experienced hematopathologist. Staining was scored as negative (−), as positive when staining intensity was similar to the staining intensity observed in large centroblasts located within germinal centers (+), and strongly positive when signals were more intense than those observed in large centroblasts (++).

### Cloning of sgRNAs

Sequences of two sgRNAs targeting *GCLC* were picked from the Brunello library (Doench et al. 2016), based on having the most prominent effect in our previously published Brunello screen. sgRNA oligos were annealed and ligated into lentiCRISPR_v2 vector (Addgene #52961 (Sanjana et al. 2014)) using the BsmBI restriction site. JM109 competent cells (Promega, Madison, WI, USA) were transformed with the ligation reaction. Plasmid DNA was isolated from a single colony using Plasmid Plus Maxi Kit (Qiagen, Hilden, Germany). sgRNA sequences were verified by Sanger sequencing (Genomed, Warsaw, Poland) Table 1.

**Table 1:** Sense and antisense sgRNA oligonucleotides targeting *GCLC*

Name	Sequence 5′ – 3′
GCLC_sg1_S	CACCGAGAAATATCCGACATAGGAG
GCLC_sg1_AS	AAACCTCCTATGTCGGATATTTCTC
GCLC_sg2_S	CACCGAGGCCAACATGCGAAAACGC
GCLC_sg2_AS	AAACGCGTTTTCGCATGTTGGCCTC

Underlined parts are compatible with the lentiCRISPR_v2 vector digested with BsmBI restriction enzyme

### Virus production

One million HEK293T cells were plated on a 6-well plate and transfected the next day using calcium phosphate transfection method (Invitrogen) with packaging plasmids psPAX (1.5 μg), pMD2.G (1 μg), and lentiCRISPR_v2 plasmid containing the sgRNA sequence (2 μg). After 24 h, 1.1 ml fresh DMEM supplemented with 10% FBS was added to the cells. Forty-eight-hour post transfection lentiviral supernatant was collected, filtered through 0.45 μm filter and used directly or stored at −80 °C.

### Growth assay

ST486 and DG75 cells were infected with two sgRNAs targeting *GCLC* gene and two non-targeting sgRNAs. Cells were selected for 4 days with 0.3 (ST486) or 3 (DG75) μg/ml puromycin and then plated out in triplicate in a 96-well plate: ST486 1000 and DG75 2000 cells per well. Next, 100 μl CellTiter-Glo reagent (Promega, Madison, WI, USA) diluted 1:2 in PBS was added per well after 1 h (baseline level), 48 h, and 96 h. The luminescent signal was measured using a GloMax microplate reader (Promega). Experiments were performed in three independent biological replicates. Growth rate was calculated at 48 h and 96 h relative to the 1-h measurement.

### BSO treatment

The effect of BSO (Sigma-Aldrich) on the survival of BL and lymphoblastoid B cells was tested at a range of 1.25–100 μM. 1 × 10^4^–4 × 10^4^ cells were plated out in a 96-well plate and treated with BSO for 48 h. Cell viability was assessed using the CellTiter-Glo assay (Promega). Survival of treated cells was determined relative to cells treated with the solvent only. The half-maximal growth inhibitory concentration (GI_50_) was calculated using GraphPad Prism 5 (GraphPad Software, Boston, MA, US). Experiments were performed in three independent biological replicates, each with three technical replicates.

### Combination therapy in BL cell lines

BL cells were plated out in 96-well plate and pre-treated with 25 μM of BSO for 24 h. The next day, either doxorubicin (Sigma-Aldrich) (80 nM for DG75; 400 nM for ST486) or cyclophosphamide (Sigma-Aldrich) (6400 μM) was added. Doses were based on the GI_50_ concentrations determined experimentally. Viability of the BL cells was measured using CellTiter-Glo assay (Promega) after 48 h. Experiments were performed in three independent biological replicates, each with three technical replicates.

### Statistical analysis

Significance of the differences in survival of cells treated with GCLC sgRNAs vs non-targeting sgRNAs and cells treated with individual drugs vs drug combined with BSO were assessed with Student’s *t*-test in GraphPad Prism 5 (GraphPad Software).

## Results

### GCLC protein is overexpressed and essential in Burkitt lymphoma

**Fig. 1:**
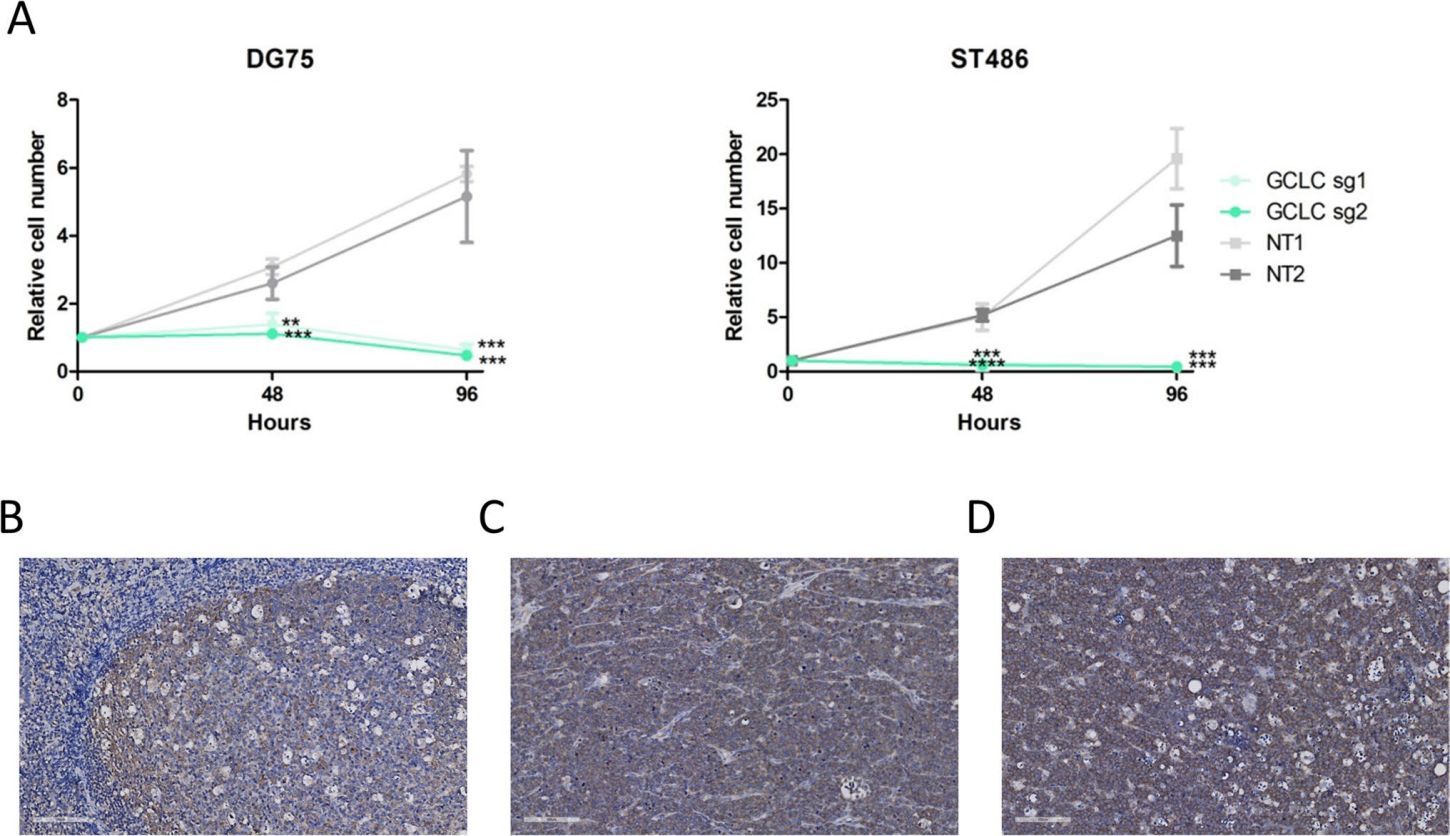
GCLC is essential for BL cell line viability and is expressed at high levels in primary BL cases. **A** Cell viability after knockout of GCLC. DG75 and ST486 cells were infected with two sgRNAs targeting GCLC. Cell viability was measured using CellTiter-Glo assay. Shown are average values and standard deviations from 3 independent experiments, each performed in triplicate. **p < 0.01; ***p < 0.001; ****p < 0.0001; *t*-test. **B**–**D** Representative example images of the staining patterns of GCLC in **B** tonsils, **C** BL tissues scored as + (positive), and **D** BL tissues scored as ++ (strongly positive)

Our genome-wide CRISPR/Cas9 screen in the ST486 cell line showed that genes encoding enzymes involved in GSH synthesis are essential for cell growth (*GCLC*: FC = −3.28, *p*_adj_ = 0.0004; *GCLM* FC = −5.3, *p*_adj_ = 5.67E-14; *GSS* FC = −5, *p*_adj_ = 0.22) (Niu et al. 2020) (Kazimierska et al. 2023). Since for GCLC protein there is a specific, clinically tested inhibitor BSO, we further focused on GCLC in our study. Using individual sgRNAs, we confirmed that *GCLC* is essential for BL cell line growth. Targeting *GCLC* in DG75 and ST486 cells strongly decreased cell viability, with a reduction of 88–91% and 97%, respectively, at 96 h (Fig. 1A). Next, we analyzed expression of GCLC in primary tissues of 14 BL cases and 3 tonsillar samples. Staining of GCLC was observed predominantly in centroblasts located within GCs in the tonsil samples. In BL tissues, staining intensity was at least as strong as in centroblasts in 6 cases and even stronger in 8 cases (Fig. 1B–D). Together, these data show that GCLC is overexpressed in BL tissues and essential for BL cell lines growth.

### Inhibition of GCLC with BSO reduces viability of Burkitt lymphoma cells

**Fig. 2:**
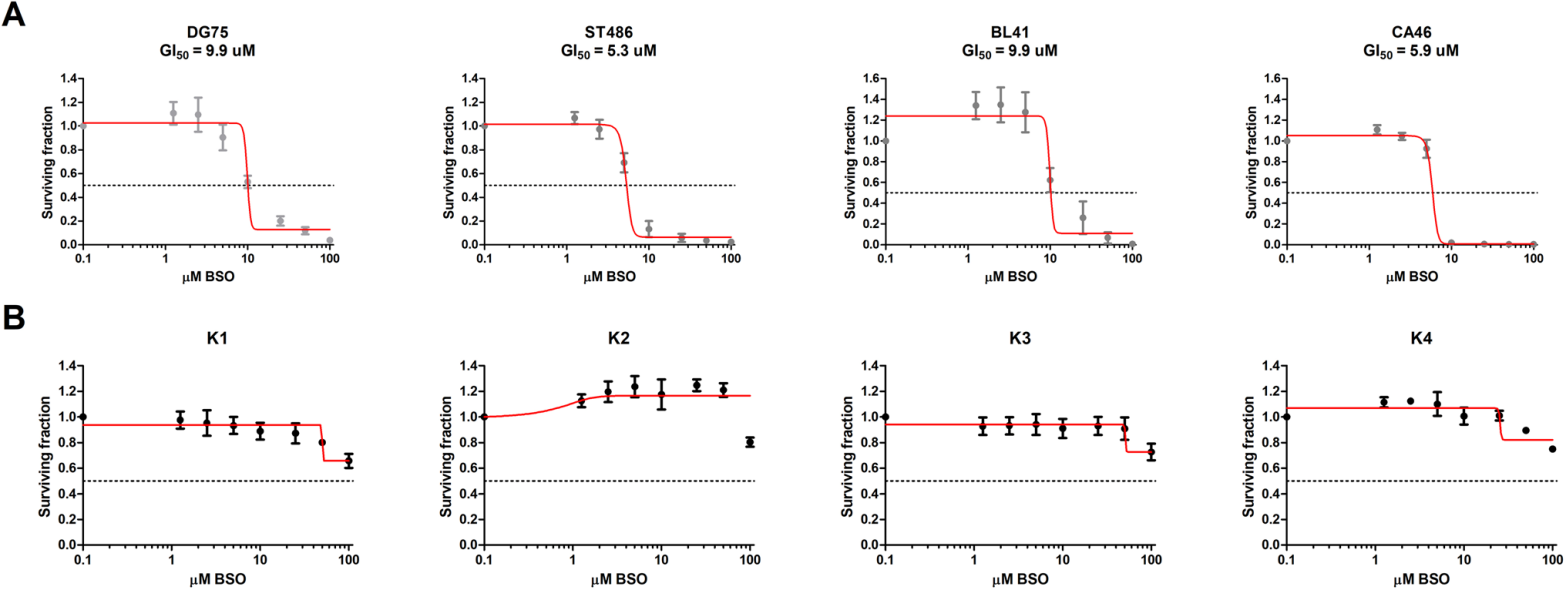
Effect of BSO on viability of BL and control lymphoblastoid B cells. **A** BL and **B** control B cells were treated for 48 h with increasing doses of BSO from 1.25 to 100 μM. Cell viability was measured using CellTiter-Glo assay, and the fraction of surviving cells relative to control cells treated with the solvent only was plotted against inhibitor concentrations. GI_50_ values were calculated using GraphPad. Shown are average values and standard deviations from three independent experiments, each performed in triplicate

We next tested if inhibition of GCLC by BSO reduces the viability of BL cell lines. We determined the half-maximal growth inhibitory concentration (GI_50_) values of BSO in four BL cell lines: DG75, ST486, BL41, and CA46. This revealed GI_50_ values ranging from 5 to 10 μM and confirmed the importance of GLCL in BL. As a control, we also treated four B-cell EBV-transformed lymphoblastoid cell lines (LCLs, K1-K4) with BSO. In contrast to BL, LCL cell lines were resistant to BSO treatment. These results show that BSO reduces viability of BL cell lines, while having no effect on control B cells (Fig. 2).

### BSO enhances the cytotoxic effect of doxorubicin and cyclophosphamide

**Fig. 3:**
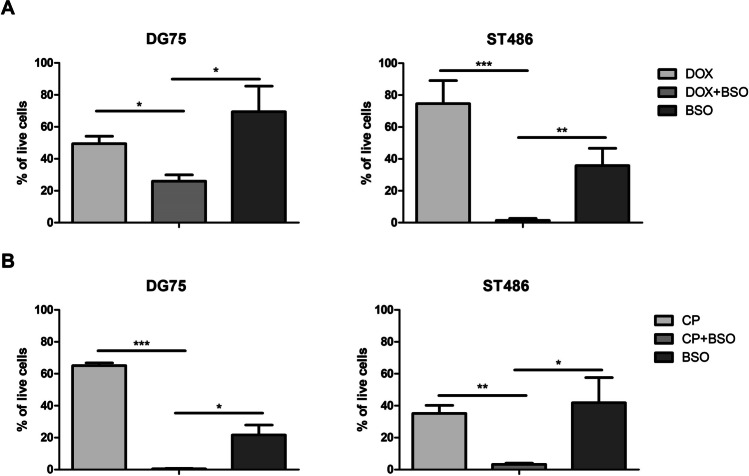
BSO enhances the cytotoxic effect of doxorubicin and cyclophosphamide in BL cell lines. DG75 and ST486 cells were pretreated with 25 μM of BSO. After 24 h, **A** doxorubicin (80 nM for DG75, 400 nM for ST486) or **B** cyclophosphamide (6400 μM) was added. Cell viability was measured after 48 h using CellTiter-Glo reagent. Shown are average values and standard deviations from three independent experiments, each performed in triplicate. *p < 0.05; **p < 0.01; ***p < 0.001; *t*-test. DOX, doxorubicin; CP, cyclophosphamide

To establish a potential beneficial effect of BSO on the effectivity of two drugs commonly utilized in BL treatment, i.e., doxorubicin and cyclophosphamide (Johnson and Abramson 2022), we tested the effect of combined treatment on BL viability. Pre-treatment of BL cell lines with BSO significantly enhanced the effect of both doxorubicin and cyclophosphamide. For doxorubicin, the percentage of viable cells decreased from 49 to 26% in ST486 and from 75 to 1.4% in DG75 (Fig. 3A). A similar effect was observed for cyclophosphamide, with a decrease in ST486 cells viability from 35 to 3% and in DG75 cells from 65 to 0.42% (Fig. 3B). These results show for the first time that pre-treatment with BSO boosts the effect of chemotherapeutics in BL.

## Discussion

BL is one of the fastest-growing human tumors. It is the most common non-Hodgkin lymphoma subtype in children. Despite improved treatment regimens, still 15–40% of patients relapse with a disease refractory to treatment. Moreover, toxic side effects, e.g., cardio- and neurotoxic effects of chemotherapy and tumor lysis syndrome, are observed in a substantial proportion of the patients. Therefore, there is a need for more specific treatments with fewer side effects. Here, we demonstrated that GCLC, the enzyme involved in GSH synthesis, is essential for BL cell lines and showed the potential of the GCLC inhibitor BSO as an anti-cancer agent.

Studies in mice demonstrated that BSO is well-tolerated and non-toxic (Dorr et al. 1986; Ishikawa et al. 1988). Furthermore, combined treatment with BSO and melphalan in xenograft models of multiple myeloma resulted in reduced tumor volume and longer event-free survival (Tagde et al. 2014). Based on the promising BSO properties in mice, phase I clinical trials with BSO and melphalan in patients with refractory malignancies were initiated in the 1990’s (Bailey et al. 1994; O’Dwyer et al. 1996). These studies demonstrated efficient depletion of GSH levels and safety of the drug. The most recent trials involved BSO and melphalan in refractory neuroblastoma. They confirmed safety of the treatment with BSO doses up to 75 g/m^2^ and achieved partial or mixed responses in 18–29% of patients (Villablanca et al. 2016) (Anderson et al. 2015). Despite these promising phase I results, no follow-up data are available about phase II trials with BSO. In a preliminary report presenting data from a trial of melphalan combined with BSO in melanoma patients, a stronger GSH depletion was observed in tumor vs normal cells (Chen et al. 1998). Recent molecular studies indicated that alternative pathways may compensate for the inhibition of GSH synthesis by BSO, such as the deubiquitinases and thioredoxin antioxidant pathways (Harris et al. 2019, 2015). Simultaneous inhibition of GSH and thioredoxins or deubiquitinases was necessary to inhibit cancer cell proliferation. Moreover, experiments in mice demonstrated that GSH inhibition with BSO can prevent cancer development if delivered before tumor onset, but has no effect once tumor has developed, potentially due to the induction of alternative antioxidant pathways (Harris et al. 2015). However, these experiments were performed in breast, lung, and ovarian cancer. Our results together with analysis of cancer dependencies available from depmap.org (Supplementary Figure 1) indicate that hematologic malignancies rely strongly on GSH and are susceptible to inhibition with BSO.

BSO GI_50_ values of BSO in BL cell lines were in the low micromolar range, while it is desirable for a potential therapeutic agent to be active in the nanomolar concentrations. In addition, the half-life of BSO is short, which would require continuous administration. Therefore, more potent and stable inhibitors could prove more effective as anti-cancer drugs. Hiratake et al. (Hiratake et al. 2002) tested a series of BSO analogues with a varying alkyl side chain for their potential to inhibit the *E. coli* GCLC. They identified several inhibitors with a more potent binding and inhibitory potential, especially sulfoximine derivatives with Et or n-Pr attached. Hamilton et al. (Hamilton et al. 2007) adopted a virtual screening of the NCI chemical database using the 3D model of human γ-glutamylcysteine synthetase (GSS). This led to the identification of four inhibitors, with structures distinct from BSO, that efficiently depleted GSH from cells, and two of them sensitized tumor cells to melphalan treatment. Although these inhibitors have not been tested *in vivo* yet, they offer a promising alternative to BSO and are worth further investigation.

Our results showed the effectiveness of BSO as a potent inhibitor in Burkitt’s lymphoma cells while having limited cytotoxicity towards control B cells. Moreover, BSO strongly enhanced the cytotoxic effect of commonly used chemotherapeutics: doxorubicin and cyclophosphamide. Our data are in line with previous work showing that BSO is not toxic to control cells and well-tolerated in clinical trials. Therefore, combination therapy with BSO could offer the potential to reduce the doses of drugs needed to be administered for an effective treatment of BL patients. Our results provide a strong rationale to further explore the effectivity of BSO and its analogues in both *in vitro* and *in vivo* experiments on a wider panel of lymphoma types.

### Supplementary information

ESM 1 (PDF 245 kb)

## Data Availability

The datasets generated and analysed during the current study are available from the corresponding author on request.
