# Supplementary material for: Inhibition of the glutamate-cysteine ligase catalytic subunit with buthionine sulfoximine enhances the cytotoxic effect of doxorubicin and cyclophosphamide in Burkitt lymphoma cells
Source: J Appl Genet. 2023 Nov 2;65(1):95–101. doi: 10.1007/s13353-023-00797-1 (PMC10789666; doi:10.1007/s13353-023-00797-1)
Supplement: Supplementary file 1 — (PDF 245 kb) [file 13353_2023_797_MOESM1_ESM.pdf]

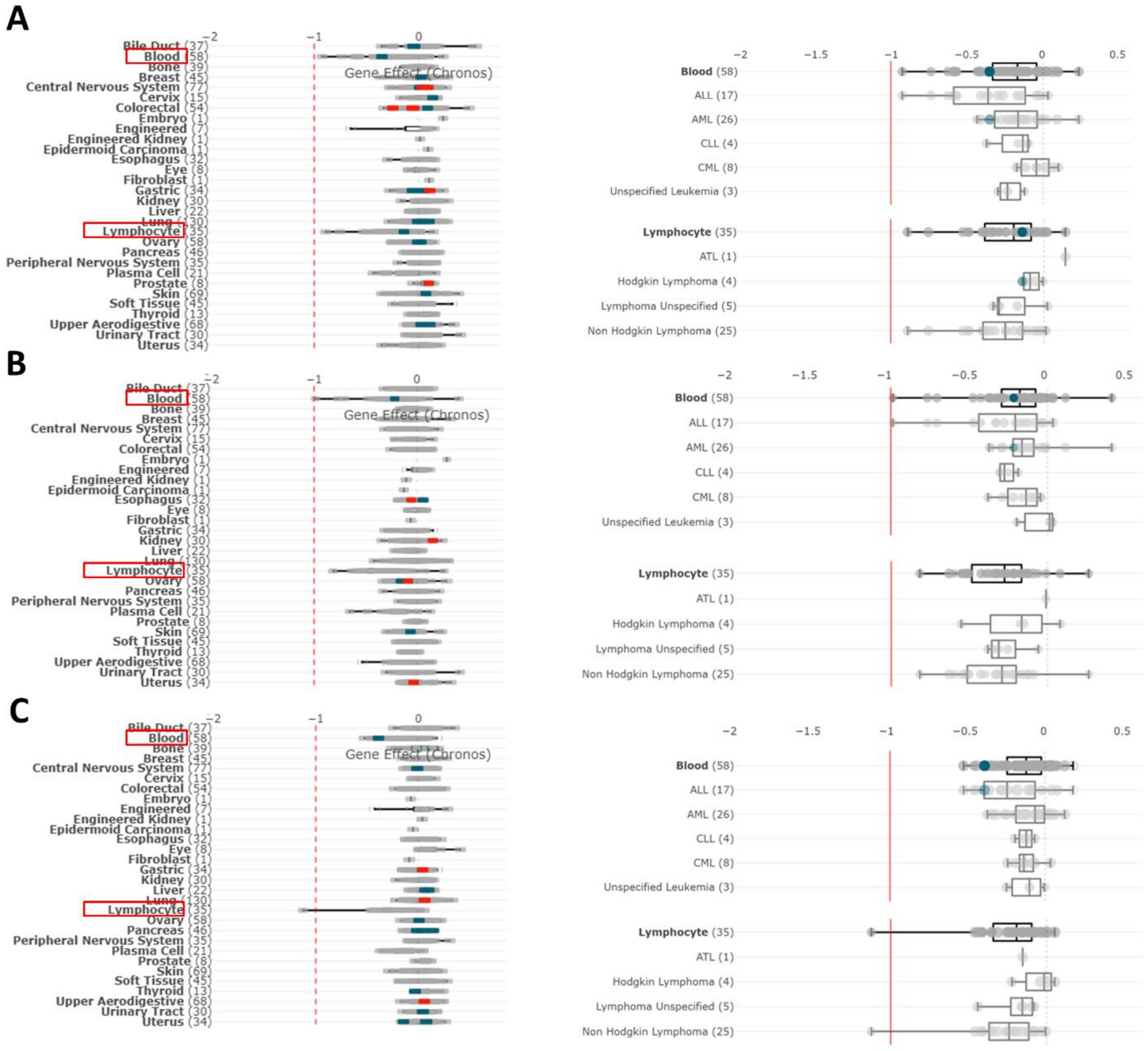

Suppl Figure 1. Essentiality of A) GCLC, B) GCLM, and C) GSS in various cancer cell lines according to depmap.org (Chronos score). Left panel – all cancer types, right panel – subtypes of hematologic cancers.
